# A Comparative Analysis of Global Datasets and Initiatives for Urban Health and Sustainability

**DOI:** 10.3390/su10103636

**Published:** 2018-10-11

**Authors:** Jonathon Taylor, Andy Haines, James Milner, Mike Davies, Paul Wilkinson

**Affiliations:** 1 Institute for Environmental Design and Engineering, University College London, Central House, 14 Upper Woburn Place, London WC1H 0NN, UK; 2 Department of Social and Environmental Health Research, London School of Hygiene and Tropical Medicine, 15-17 Tavistock Place, London WC1H 9SH, UK

**Keywords:** urban, health, sustainability, data, organizations

## Abstract

Globally, urban populations are growing rapidly, and in most cases their demands for resources are beyond current limits of sustainability. Cities are therefore critical for achieving national and international sustainability objectives, such as greenhouse gas reduction. Improving sustainability may also provide opportunities for urban population health co-benefits by reducing unhealthy exposures and behaviours. However, there is currently sparse empirical evidence on the degree to which city characteristics are associated with variations in health-related exposures, behaviours and sustainability. This paper examines the feasibility of aggregating empirical data relating to sustainability and health for global cities. An initial scoping review of existing English-language datasets and networks is performed. Resulting datasets are analysed for data types, collection method, and the distribution of contributing cities across climates, population sizes, and wealth. The review indicates datasets are populated using inconsistent methodologies and metrics and have poor overlap of cities between them. Data and organisations tend to be biased towards larger and wealthier cities, and concentrated in Europe and North America. Therefore, despite vast amounts of available data, limitations of reliability, representativeness, and disparate sources mean researchers are faced with significant obstacles when aggregating data to analyse the sustainability and health of globally representative samples of cities.

## 1 Introduction

The world is currently undergoing a period of rapid urbanisation, with the global urban population overtaking the rural population in 2007, and this trend set to continue into the future [1]. The urban population is predicted to grow by an additional 2.5 billion people by 2050, with growth particularly high in Africa and Asia [1]. Due to their population share, economic influence, and resource requirements, cities are likely to play a key role in achieving national and global sustainable development targets. This rapid expansion of cities—in terms of number, population size, and built area—presents challenges as well as opportunities to influence their sustainable development at nascent stages in their growth. Improved sustainability can have co-benefits for population health, with urban development an important determinant of non-communicable disease burden through its effects on physical activity, food choices, social interactions, exposure to air pollution, crime,and extreme events [2]. Urban policies are therefore an important driver of population health and wellbeing—but the potential is often unrealized [3].

The critical need to improve sustainability is reflected in a vast number of global, national, and local initiatives. At the international level, the United Nations Sustainable Development Goals (SDGs) reflect a broad consensus on the need for an integrated approach which addresses the social, economic and environmental pillars of development. The SDGs include goals related to urban areas, in particular SDG 11, which aims to “make cities and human settlements inclusive, safe, resilient and sustainable by 2030”. SDG 11 includes 10 targets, defined using national-level indicators that range from internationally standardized metrics with readily available data, to as-yet unstandardized metrics using data that is not yet universally available, and tracking these metrics at a sub-national level is critical to evaluate progress. Due to the importance of urban areas in achieving sustainability goals, there are ongoing initiatives to localise the SDGs, defined as ‘adapting, implementing, and monitoring SDGs at the local level’ [4]. The need to improve urban sustainability and population health is also reflected in large number of global and national city networks, organisations and research projects dedicated to promoting collaboration, sharing information, and collecting data on sustainable development.

However, despite global recognition of the urgent need to improve sustainability, and the growing number of initiatives promoting city-level sustainability and health, there is patchy empirical evidence that demonstrates how city characteristics and policies are associated with sustainability—for example Greenhouse Gas (GHG) emissions, population behaviours, exposures to environmental hazards such as air pollution, and health outcomes. This has led to an increase in research termed ‘epidemiology of the urban’ [5], where the causation of urban health and sustainability problems is investigated using collections of city-level data and metrics. Such research requires reliable, open, clearly defined, and globally representative data to inform the best methods for achieving sustainable and healthy development for cities around the world, which are all developing under their own unique set of environmental, economic, demographic and political constraints. Data to support analyses may be obtained through (1) experimentation, particularly diverse projects implemented across a range of contexts, and strengthened assessment of project outcomes [6], or (2) collected from existing surveys, academic publications, and datasets held by government and non-profit organisations. This data is critical in order to compare cities, to help evaluate their policy performance, and to help shape development pathways that promote sustainability and health.

As part of the Sustainable Healthy Urban Environments (SHUE) project, we have examined the feasibility of aggregating existing, open, and off-the-shelf data from various sources to enable such global analyses, and the obstacles to doing so. First, we carry out an initial scoping review of existing international city-level initiatives involved with health and sustainability (defined here as non-governmental or international governmental organisations that seek to promote collaboration and knowledge sharing in these areas between cities). We then extend the review to seek existing published global datasets that provide information on city-level sustainability characteristics and population health. This paper reports the results of that review, including the current data and organisational landscape. We then perform a series of analyses to examine the representativeness of cities within these initiatives and datasets to evaluate any population, wealth, climate, and geographical biases and to identify the degree of overlap of constituent cities. Based on these analyses and the obstacles faced when aggregating the collected datasets, we suggest potential forward paths for collecting data that may be used to evaluate the associations between health, sustainability, and environment in cities to provide a stronger evidence base for policy decisions.

## 2 Materials and Methods

### 2.1 Review

For this study, we focus on environmental sustainability, particularly related to the consumption of natural resources, transport, housing, energy, diet, policy, urban environment, and basic measures of economic wealth. For urban health, we include population health, as well as additional factors known to influence urban health such as air pollution, climate, and disaster risk. A scoping review was performed to identify existing datasets and organisations involved in in the above areas. We initially searched for search terms (Table 1) within academic abstracts on the Web of Science). Results were filtered by reading the abstract/introductory text to determine if the study met the inclusion criteria. The inclusion criteria were defined as:

1. Only datasets and organisations with information in English were included.
2. Datasets and organisations must include cities from more than one world region (Europe, Asia, North America, South America, and Australasia). This was done to (a) exclude the vast number of federal datasets available, and (b) attempt to somewhat overcome the limitations of searching only in English by looking for global initiatives only.
3. Only those with greater than 10 cities were included. This was to limit the results in order to focus the analysis only on larger datasets that are more likely to be globally representative.
4. Only those with openly available lists of contributing cities were included; those available with open data or through academic subscriptions were included, while all commercial datasets and datasets where access needed to be requested were excluded.
5. As many cities have changed rapidly in the past decade, only data (published after 2010) was considered.
6. We searched for indexes, but only to identify sources of underlying data. We did not include indexes themselves in the analysis as their calculation can have a lack of transparency and can include methodological biases [7].
7. We excluded gazetteers, where information was limited to attributes such as population, location, and country.

For both organisations and databases, results were summarised in terms of their scope, and the cities—and number of cities—contained within them. Additionally, for datasets, the variables were collected. The references of papers that met the selection criteria were searched for additional datasets and organisations.

As multiple known datasets did not appear in the search of the academic literature, we repeated the search in two grey literature databases, Open Grey [8] and Grey Literature Report [9] using the same structured search terms and exclusion criteria. When this did not produce additional results, we sought to identify additional organisations and data by searching using a semi-structured Google web search, looking through the top 100 results for the various combinations of search terms in Table 1. Here, we investigated websites where the descriptive text suggested that the hit was relevant to our study. Duplicate organisations or datasets—or those that had already been found in the previous searches—were excluded. While issues with data reliability and consistency were apparent, we did not filter the results using any tools that assess data quality, but did assess potential biases using the methods described below.

### 2.2 Analysis of Data

An analysis of the databases was performed in order to examine their scope and coverage of data. This included reviewing the scope and number of cities held within each dataset, and the data availability. All open-source datasets were downloaded, and an analysis performed to examine the types of data held within them. The parameters held in each dataset were classified according to whether they were related to demography, geography, transport, economy, energy, environment, policy and government, health, and housing. Heatmaps were created in SAS 9.4 [10] for each database that showed the numbers of cities and metrics available for each data classification within each of the databases, and the amount of overlap between the datasets was quantified.

To examine geographical, size, climate, or wealth biases in the different available datasets, cities with each organisation or database were compared against Geonames [11], a gazetteer that integrates data from several sources including the National Geospatial-Intelligence Agency’s (NGA) and the U.S. Board on Geographic Names. The Geonames dataset was assumed to represent the best available source of global distributions of all cities. All cities within each of the identified datasets were provided population and geolocation information using the Geonames Application Programming Interface (API) that queried the Geonames database using the city name, local area, and country. Results were checked manually to ensure geoclassifications were accurate. The city coordinates were used to locate cities within Baileys Ecoregion domain [12] using ArcGIS [13]; ecoregions are used here as a proxy for regions of related climates differentiated by temperature and precipitation (dry, humid temperate, humid tropical, or polar), which may influence both population health, behaviours, and city sustainability characteristics. In the immediate absence of information on city-level wealth, we used the gross national income (GNI) to indicate city development according to the World Bank income classifications for 2015 (Low: $1045, Lower Middle: $1045–$4125, Upper Middle: $4126–$12,746, High: >$12,746). Cities were also classified by population (0–99,999, 100,000–499,999, 500,000–999,999, 1,000,000–4,999,999, and >=5,000,000) using the population data held within Geonames.

The resulting information was analysed to explore the availability and representativeness of databases, indices, and organisations across different ecoregion, population, and wealth classification using SAS relative to all Geonames cities. The number of initiatives to which cities belonged to were summed, and used to develop a kernel density heatmap in ArcGIS to show global variations in data availability and organisational membership. A heat map with all Geonames cities was also created to contrast global city density with organisational and data density.

## 3 Results

### 3.1 Review

**Figure 1:**
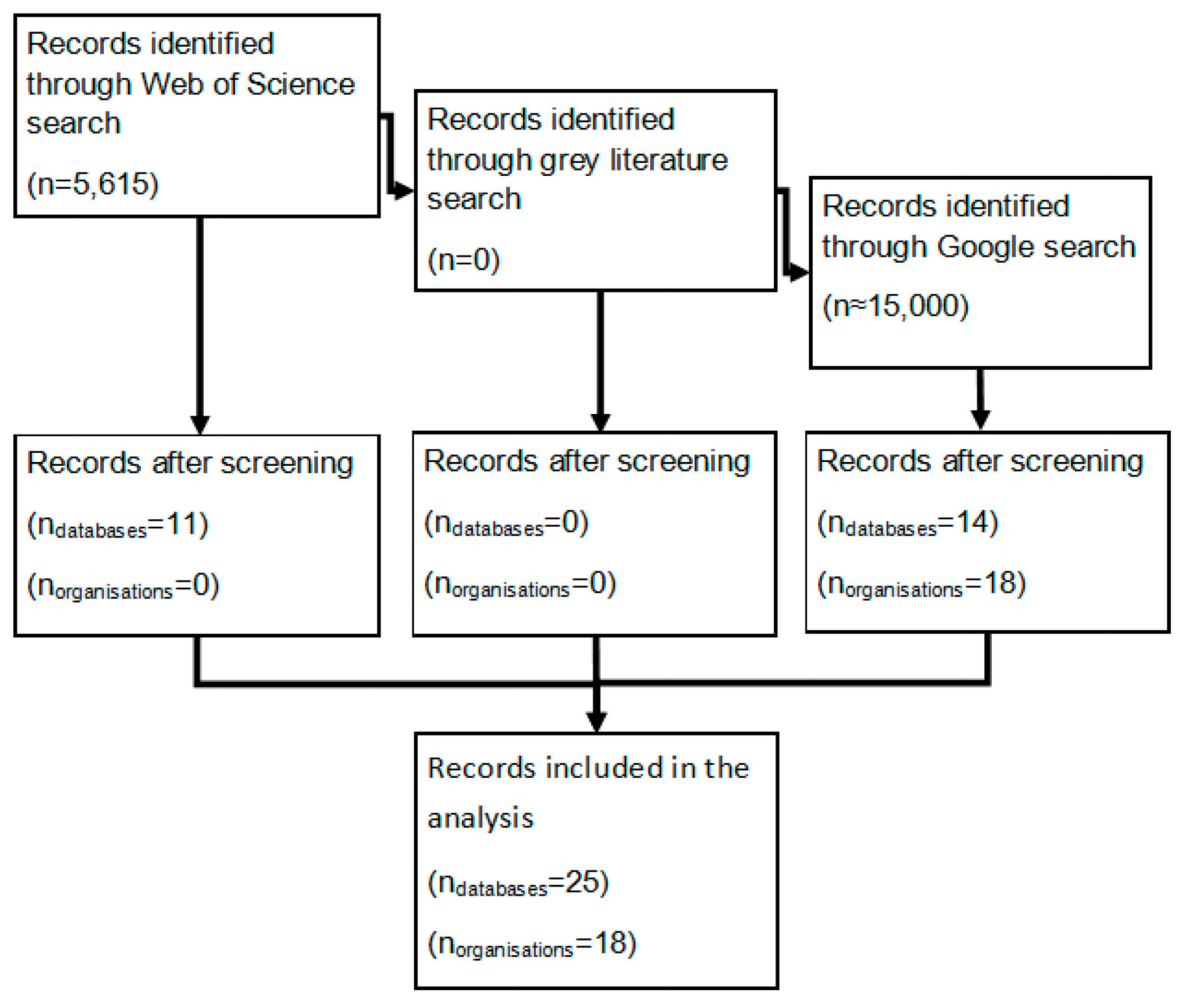
Flowchart indicating the selection of relevant organisations and datasets.

The process of identifying relevant organisations and datasets is summarized in Figure 1. The search terms led to 5615 results on Web of Science. Following screening and application of the inclusion criteria, these papers were subsequently reduced to 11 papers that referred to relevant, unique and available datasets that met the selection criteria. Several papers were found that described the generation of global gridded data that may be used to inform city characteristics, or that described the analysis of data from existing commercial datasets; these were excluded, but are referred to in the discussion. Searches in the grey literature databases were unable to produce any relevant results. The search on Google produced a large number of results, which, after screening and the removal of duplicates, left 32 relevant initiatives. All results are summarised in Tables 2 and 3.

#### 3.1.1 Organisations

The review indicated 18 global organisations that promote the sharing of information and collaboration between local governments, with a focus on urban health and sustainability, listed in Table 2. These organisations may be classified as networks of cities, or organisations that work to foster collaboration between cities, and are funded through private initiatives, memberships, and through intergovernmental organisations. Due to the significant overlap in scope, several partnerships and links exist between many of these organisations. In other cases, organisations have merged, such as the Covenant of Mayors [14] and Compact of Mayors [15] to form the Global Covenant of Mayors for Climate and Energy [16]. Some of these organisations—such as CDP (formerly the Carbon Disclosure Project) [17], C40 [18], and the Global Cities Indicator Facility [19]—also make data available publicly to researchers (see Section 3.1.2 below). Also included en Table 2 are seven organisations that facilitate or coordinate action on sustainability and health at the urban level, but which are not composed of city networks; these have been excluded from further analysis.

#### 3.1.2 Databases

Our focus was on data collected, collated, or derived by organisations that are publicly available; we excluded the multiple databases that are commercially available, as well as any source that did not provide the data in the document text, appendix, or in a related web-based data repository. The review identified several different datasets, produced by academics, governments, and non-governmental organisations (Table 3). These datasets could be classified as official or unofficial data collated by organisations, or data derived or modelled for cities based on underlying data acquired individually for each city. Collated data is collected using several methods, including: city governments applying for membership and submitting data; aggregations of data published by individual cities, organisations, or in research papers; collected through individual initiatives such as urban observatories satellite image analysis; or various combinations of the above.

There are several datasets which provide large amounts of aggregated city-level data on health and sustainability. These include those from various UN sources, including UNData [39], UrbanInfo [40], and the UN Data Explorer [41], as well as the OECD [42], the NY City Global City database [43], the WHO Air Pollution [44], and World Bank Urban Transport Data Analysis Tool (UT-DAT) [45]. One limitation of city-level datasets is the variation in the ontology and definition of metrics reported for different urban areas. This is particularly problematic in the case of some of the UN datasets, where data has been submitted or aggregated from local or national governments censuses or from local urban observatories; issues include variations in the definition of the urban area, different metrics, and different methods of calculating these metrics.

Membership-based organisations require cities to apply to join, and then regularly submit information to track city progress. Examples of such datasets include those provided by the CDP [17] and its partner C40 Cities [18], which make data on greenhouse gas emissions and policy available through an open data portal. Similarly, the Global Cities Institute [19] provides data for a selection of cities via the World Council on City Data [46]. In addition, the Global Cities Institute [19] requires data be submitted using standardised metrics defined by ISO 37120 [47], which means that data is comparable between cities, overcoming a significant limitation faced by users of other datasets. However, organisations that require submitting data often have the goal of supporting city cooperation rather than research, and they do not make all data public. They also require cities registering and submitting data, which may be dependent on cities having the wealth, available staff, systems and willingness to do so.

Modelled or derived data is available for cities describing their built environment, population health, projected future characteristics, and financial performance. While there is a general lack of data related to health at the city level, epidemiological studies such as the PURE study [48] and the work by Guo et al. [49] describe the health outcomes given population behaviours or temperature exposures for several cities worldwide. Other studies reviewed large numbers of studies to derive datasets [50–54]. Another source of derived city information is remotely sensed satellite imagery, which was used to assess city growth [55,56] or land use [57] and is available for a sample of cities.

Databases such as the Atlas of Urban Expansion (AoE) [55] and the UN Sample of Cities avoid biases from self-reporting or aggregating by randomly sampling urban locations from around the world, and then obtaining data from these locations. These datasets employ a global, stratified sampling technique to select their cities. For the AoE, originally 120 cities were sampled from 4231 global cities with a population of 100,000 or more, stratified according to the world region in which the city is located, the city population, and GNI per capita. This selection has since been expanded to 200 cities, and provides the foundation for the UN Sample of Cities. This approach provides the advantage of having a statistically representative sample of cities for cross-city analyses, and is possible largely due to the fact that the data within these datasets is derived using open source geographical information.

In addition to databases of single indicators, several databases containing calculated indices of performance on several different themes were identified. As with the above databases, these may be provided by commercial, non-governmental, or academic sources. While potentially valuable sources of information and perspective, there are several issues with such indices, including a lack of transparency and potential methodological biases in many indices [7], particularly those where calculation methods are hidden to readers. Consequently, we have not included them in this analysis, but refer readers to previous reviews [58].

### 3.2 Analysis of Organisations and Databases

#### 3.2.1 Organisations

**Figure 2:**
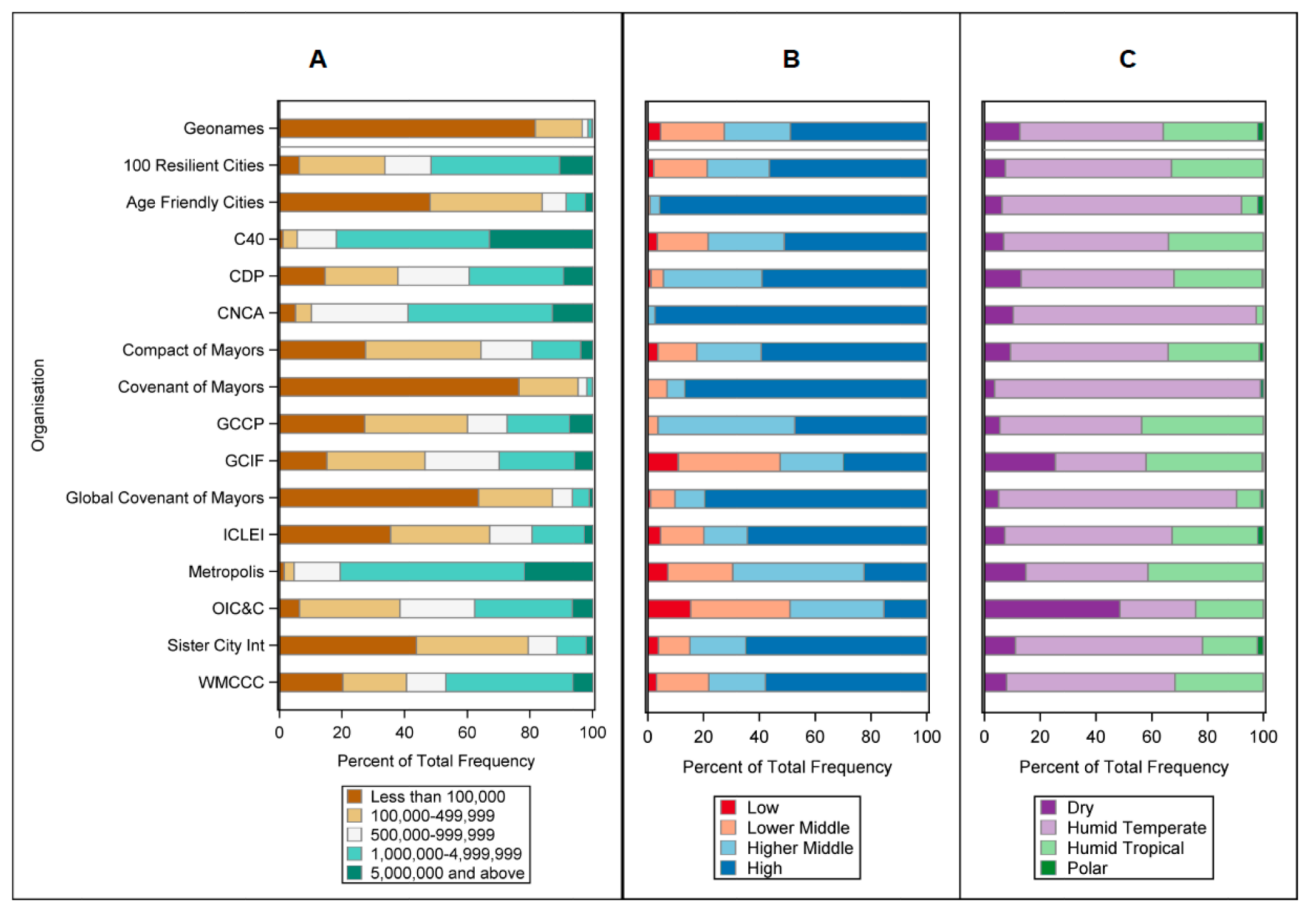
The distribution of membership cities by organisation across different (**A**) population sizes, (**B**), GNI, and (**C**) ecoregion clasaification. Refer to Table 2 for acronym definition and organization details.

Figure 2 shows the distribution of organisations listed in Table 2 with publicly available member cities, across different population size, wealth, and ecoregion classifications (lists of member cities were not available for CLGF Safer Cities, and UCLG). The Geonames dataset provides an estimate of the global distribution of urban environments, against which databases are compared in order to evaluate potential biases. Organisations tend to be over represented in wealthier countries, in temperate regions, and those with larger population, indicating that the cities that participate in such organisations are not necessarily globally representative. All organisations differed significantly in terms of population distribution from Geonames, as our definition of urban area included those with populations as low as 15,000. It should be noted that there are several potential biases in the underlying Geonames dataset, with previous analyses suggesting greater densities of cities in Western Europe and the United States, and lower densities that expected in Africa and Asia [65]. Of the 9910 cities involved in the various organisations, the most widely involved were Vancouver (15 organisations), Toronto, Melbourne, Miami, Barcelona, and Paris (13), and Seoul, Quito, Oslo, and Berlin (12).

#### 3.2.2 Databases

**Figure 3:**
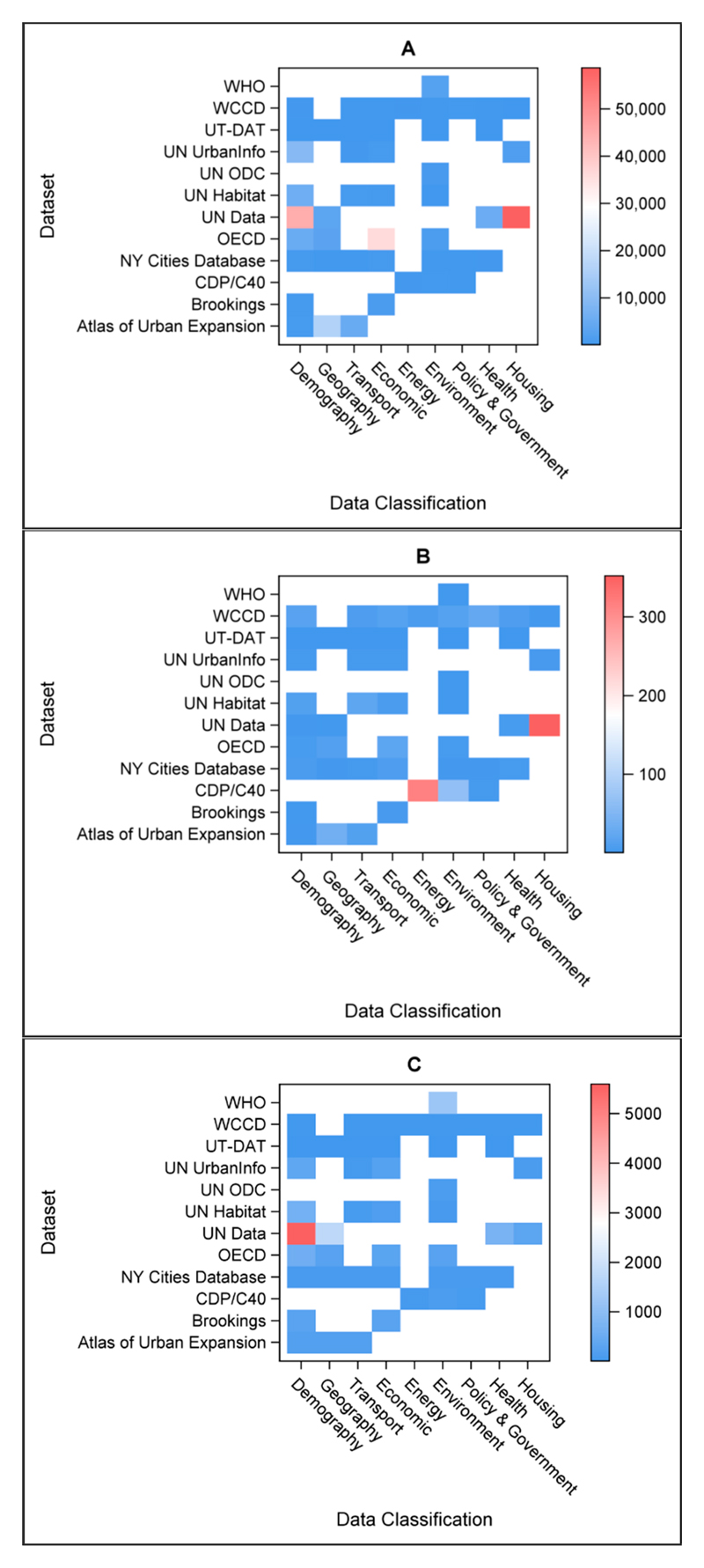
A heatmap showing the amount of data available in the open datasets, for (**A**) all data—both cities and metrics, (**B**) the number of metrics, and (**C**) the number of cities, by different category. Refer to Table 3 for acronym definitions and details.

The availability of data within each of the datasets with openly available data can be seen in Figure 3, for all data (cities and metrics; (A)), the metrics reported (B), and number of cities (C). This shows the range and availability of data from each datasource, and demonstrates, for example, a focus on collecting a large number of metrics for a small number of cities, or vice versa. A matrix is shown in Table 4 that indicates the degree of city overlap between the different databases. Of the 6637 unique cities contained in the datasets, London occurred the most frequently (24), followed by Tokyo and Hong Kong (22), Madrid, Paris, and Seoul (21), and Beijing, Berlin, Los Angeles, and New York City (20). Only 27% of the 6637 cities found in the combined datasets occurred more than once, demonstrating a low level of overlap between data sources.

**Figure 4:**
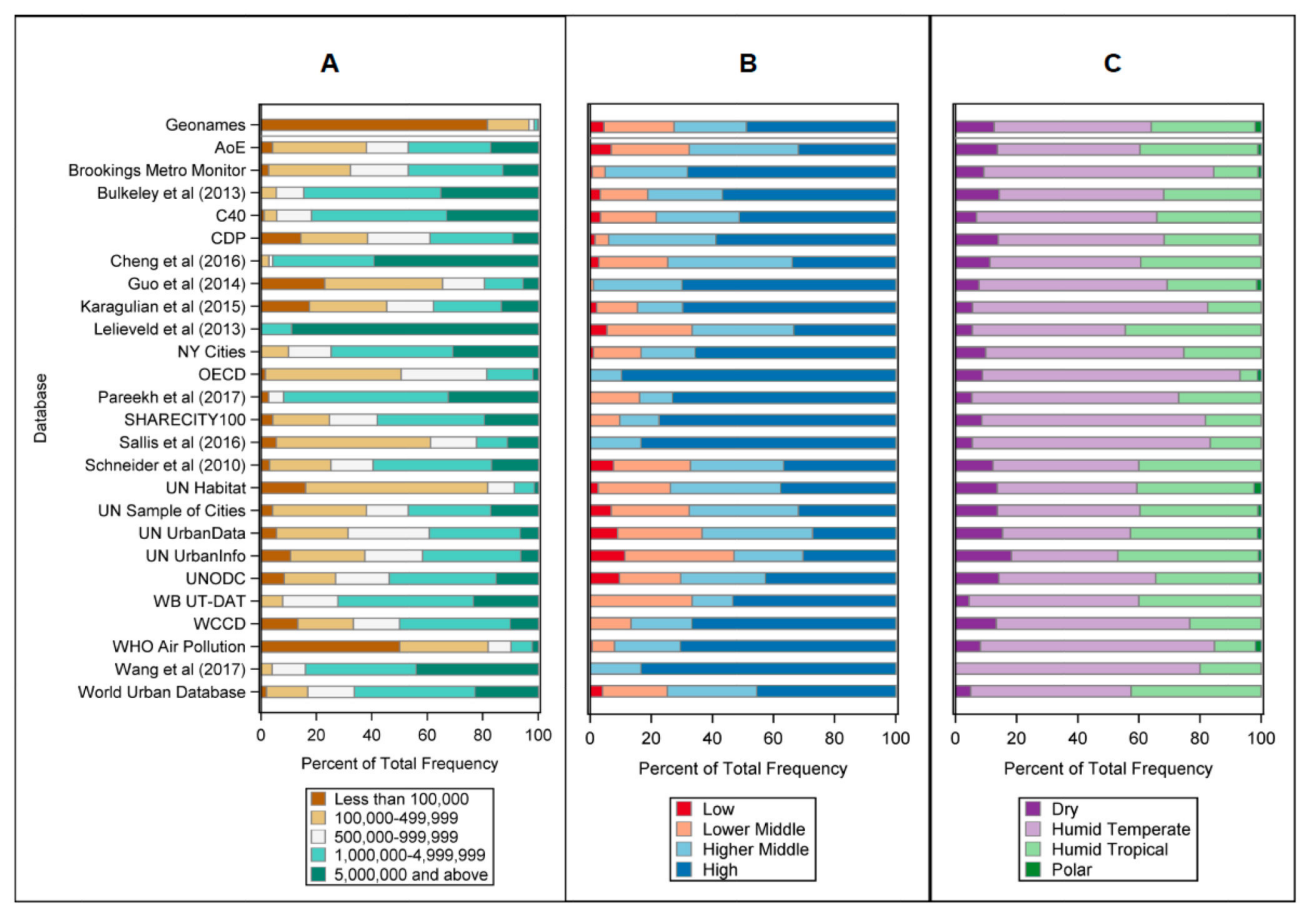
The distribution of cities contained in datasets according to (**A**) population size, (**B**) GNI, and (**C**) Ecoregion. Refer to Table 3 for acronym definitions and details.

Figure 4 shows the distribution of cities in all the databases in Table 3, according to population size classification, GNI, and ecoregion. Many datasets are biased towards temperate regions, and those with higher GNI classifications, suggesting that cities within these databases are not globally representative samples. In many of the datasets, biases are inherent to the types of organisations that collect the data, such as the bias towards high income cities contained in the OECD dataset. As with the organisations, there is a significant difference in the population distributions between the Geonames ‘universe of cities’ and the other datasets.

### 3.3 Spatial Analysis

**Figure 5:**
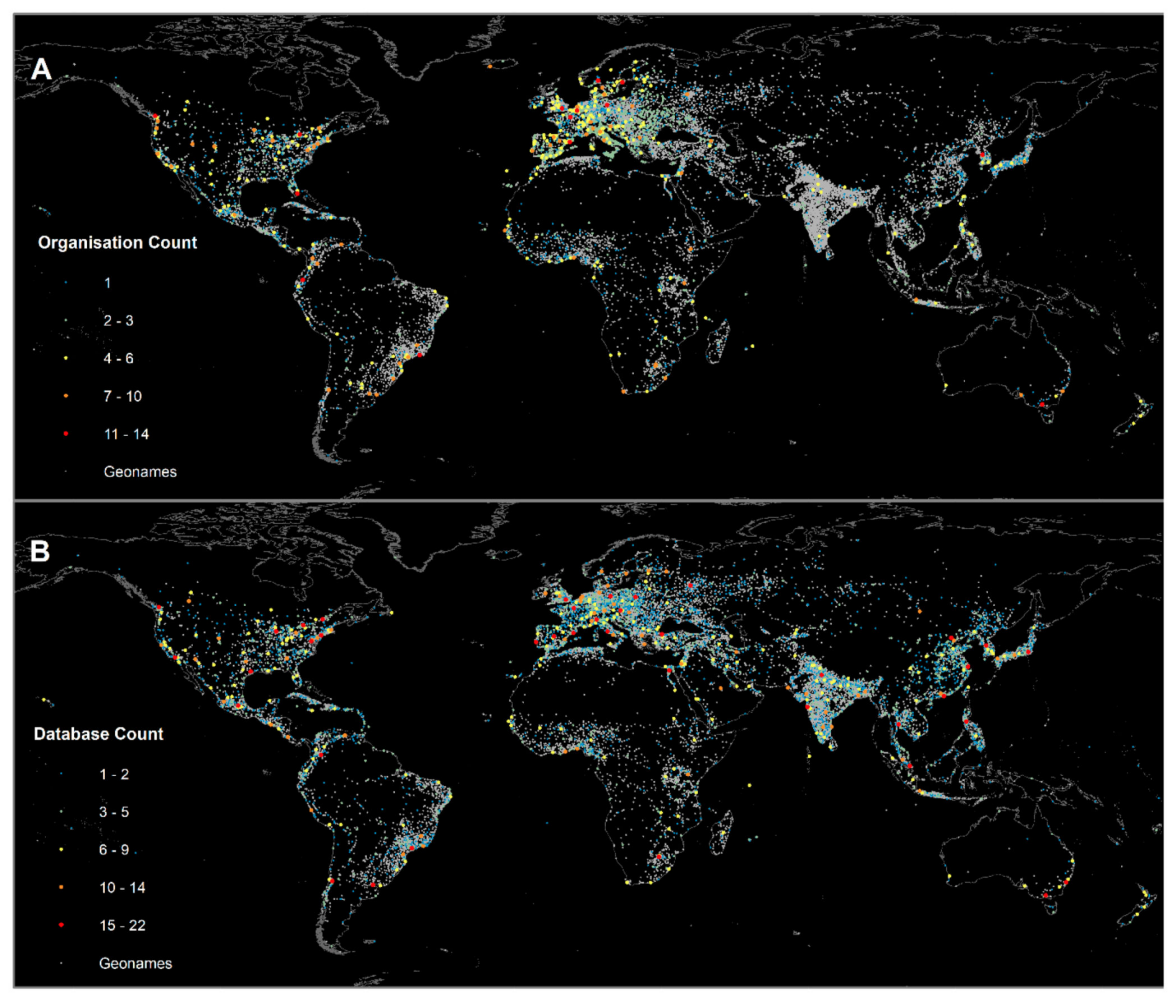
Heat map showing the density of cities with (**A**) organisational membership, or (**B**) inclusion in databases. These overlaid on top of cities in the Geonames database with a population over 15,000.

The spatial variation in organisational membership and data availability in contrast to Geonames city density can be seen in Figure 5A,B, respectively. For the analysis, we excluded organisations that were subsidiaries or components of larger organisations so that cities were only counted once. The underlying Geonames data has been previously shown to underestimate the number of urban areas in Asia and Africa [65]; however, the API was able to provide a near complete match rate of for cities in the different organisations and databases. Both organisations and databases are also underrepresented in East Asia and Africa, meaning that the discrepancy between urban density and organization or data membership is stronger in these areas than is indicated in Figure 5.

## 4 Discussion

We have performed an initial review of organisations and databases that are involved in sustainability and health at the city level, and presented an analysis of the cities within them across different wealth, climate, population classifications, as well as their geographical location. We have also analysed the parameters held within the databases to demonstrate their particular focus and quantified the degree of overlap between them.

### 4.1 Obstacles to Aggregating Existing Data

The review provides a useful summary of the key globally available datasets relating to health and sustainability; however, the above results indicate several significant obstacles when collating city-level data from existing open sources in order to investigate the relationship between city characteristics, sustainability, population exposures and behaviours, and health. These raise some important issues that make aggregating existing ‘off the shelf’ data for urban health and sustainability analyses difficult in practice, which are discussed below.

#### 4.1.1 Identifying Relevant Data and Organisations

Our scoping review of existing city-level organisations and datasets found several initiatives to collect information and benchmark city performance in relation to urban health and sustainability. The review was intended as an initial investigation into datasources, the cities represented within these datasources, potential biases by city types, the metrics collected, and obstacles faced when aggregating the data and using it to perform analyses.

During the review, few results were found in the academic and grey literature sources, missing many that we were aware of prior to beginning the search. This may in part be due to limitations in the search strategy—we only searched a single academic publication database, and with relatively broad search terms. The search on Google was much more successful in finding relevant initiatives, but required a more simplistic use of key terms than in the search of academic and grey literature, and may not be easily replicated. In addition, it was not possible to search every page of the returned Google searches. As such, the multiplicity of data available means that this review is unlikely to be comprehensive, but can be improved on and refined in the future. It also raises a key obstacle to this work—relevant datasources may not be easily found, may only be available in non-traditional searches, and in many cases may require prior knowledge of their existence to be able to find.

The international databases and organisations that have been reviewed and included in the analysis are those which are available in English, which was a necessity due to it being the language of the study authors. This is likely to be another significant source of bias- there are a number additional networks—such as the Union of Ibero-American Capital Cities (UCCI) and the Association internationale des maires francophones (AIMF)—that boast large networks of global cities, but that were not included in the analysis. Regional, federal, or individual-city datasets were also not included in the review and analysis. Identifying the relevant national or local datasets, and aggregating the data to a common format, would add a significant time cost when developing datasets for analysis.

#### 4.1.2 Coverage of Data and Organisations

The analysis identified inconsistencies in data and organizational coverage between different population, wealth, and climate categorisations, as well as the geographical coverage. Much of this is likely to be due to the necessary inclusion criteria of the review, and coverage is likely to improve if non-English and regional/federal/city-level databases were to be included. Indeed, some of the reviewed organisations and datasets examined have developed organically and are not intended to be globally representative, while others such as those from the OECD are by their nature limited to certain countries. However, the analysis suggests that wealthier cities have greater involvement across multiple organisations, which may have implications for policy formation if data from these sources are used to inform policies in low/middle income settings.

Our analysis of organisations and datasets was done relative to a global universe of urban areas with populations greater than 15,000, in order to include smaller cities that according to Geonames compose around 80% of the global urban population, and are the urban areas where population growth is predicted to be greatest [1]. There are also significant opportunities to influence future sustainable and healthy development in nascent cities by making key early planning decisions. However, these cities are under-represented among the organisations and little data is available on them. This is another significant obstacle, as gathering more information on these smaller cities will be critical for informing and evaluating sustainable development. There are also issues with defining the ‘universe’ of cities with which to obtain representative samples. As mentioned previously, biases exist in the underlying Geonames dataset used to define the global distribution of cities, with lower than expected densities of cities in Africa and Asia [65]. We used Geonames as it is the largest gazetteer, and is likely to offer the greatest coverage of global city data. However, the universe of cities used here is not representative of global cities, which has implications for selecting truly ‘representative’ samples, and has led to a bias in the underlying dataset with which the reviewed datasets and organisations are compared. This is particularly evident in Figure 5; there is a low density of organisations and data from cities in Asia and Africa, and the low density of Geonames cities means the actual bias may be greater than our analysis suggests.

In addition, the analysis indicates a low level of overlap between cities participating in different organisations, and the cities with data held on them in the various databases. While there was a degree of overlap between individual datasets (Table 4), overlap between multiple was poor. This presents obstacles when trying to aggregate data from various sources into a single dataset, as there may be few common cities, and reduced the probability of being able to derive globally representative datasets with data collated from different sources. There are key differences in temporal coverage across the various datasets, with the data recorded at different time intervals, and while some gaps may be filled using interpolation techniques, in some cases city data was not available for the recent past. In general, there is very poor availability of city-level health data globally, which presents obstacles when quantifying health impacts of urban environmental exposures or behaviour changes. There is therefore a need for greater city-level health data for comparative analysis [66], and to enable greater linkage between city environmental characteristics, policy, and health outcomes.

#### 4.1.3 Data Reliability

We found that the definition of a ‘city’ differed between datasets, and occasionally within datasets, with some referring to the city as defined by an administrative boundary, others referring to the wider metropolitan area or built-up area; metadata provided with the data often did not clarify what the number alluded to. As has been noted previously [47], there are a wide range of different metrics and data gathering methods both within and across different datasets, which means that values may not be comparable between cities, or that a conversion between metrics is necessary. While we have not evaluated the quality of data held within each dataset, it is apparent that data errors and inconsistencies are present. The lack of results from the search of academic publications means that much of the data has not been peer reviewed, and the degree of quality assurance is often unknown.

#### 4.1.4 Data Accessibility

Some studies found during the search but excluded from the final analysis were found to reference commercially available datasets, which they had licensed. There are a large number of such datasets provided by different organisations; these have not been included in the review. In other cases, data was only available through an academic subscription, or through cities that register to the different organisations. Without the data being openly available, it is potential for use by researchers is limited. While many data providers offered opportunities to download the data in various file formats—including non-proprietary text formats—the structure of the datasets were often different, and arranging the data to fit within the database schema requires further work. Metadata was often unavailable, and with some metrics there is a lack of transparency in the calculation methods.

### 4.2 Other Datasources

The data sources identified above may be limited in their usefulness as tools for analysis, as they are (1) rarely globally representative, (2) often contain non-standardized metrics, or contain a limited number of indicators, and (3) have poor overlap between participating cities. The individual datasets above do address these issues, but not currently all three.

While not included in this review, there are several additional sources of non-aggregated data which may be used to inform city characteristics or derive relationships between health, sustainability, and environment. The data from several national census are available to download via HTTP requests, allowing data acquisition to be automated. Without standardization of metrics, however, this can lead to many of the data reliability issues mentioned above, and some degree of user curation is still likely to be required. Similarly, data scraping from online sources, such as Wikipedia, may offer opportunities to obtain data for large numbers of cities; however, this approach may also offer several potential reliability issues, and the necessary metadata may not always be available.

Raw data sources may provide valuable information for exposure data. For example, weather data for global locations is available from services such as the UK Met Office Weather Observation Website (WOW) [67], the Met Office Integrated Data Archive System (MIDAS) [68], and Wunderground [69]. Monitored real-time air pollution measurements are available from sources such as Air Quality Index China (AQICN) [70] and BreezoMeter [71]. Future climate projections are available from services such as Hothaps Climate CHIP [72].

Geospatial data may also be used to extract relevant data for cities. Modelled datasets that may be spatially matched to cities include global climate models, which may provide important climatic variables under current and projected future conditions (e.g., [73]), natural disaster data [74], and emissions estimates [75]. Geographical data sources such as WorldPop [76] may also be used to acquire local data for cities. Remotely sensed, modelled, and Geographic Information System (GIS) data is available for cities from several different sources. Such datasets enable the extraction of city data based on coordinates or city outlines, and if sufficient resolution may offer the opportunity to examine intra-city variation of data. Examples of remotely sensed data includes imagery from the Advanced Spaceborne Thermal Emission and Reflection Radiometer (ASTER) satellite [77], including landcover and land surface height (via a Digital Terrain Model), which may be used to analyse vegetation, built-up area, and hilliness within the city extents. Similarly, satellite-derived air pollution data [78] and land surface temperature data (demonstrating Urban Heat Island increments) may be acquired for cities [79]. Advantages of such remotely sensed data include the ability to acquire temporally varying data and monitor changes over time. Tools such as Google Earth Engine [80] may be used to automatically extract data, given defined city boundaries. GIS datasets such as OpenStreetMap (OSM) [81] may be able to provide data on road and transportation networks, water bodies, and land use for areas within the city boundaries, depending on the availability of OSM data for certain locations.

### 4.3 Ongoing and Future Work

The Sustainable Healthy Urban Environments (SHUE) project has combined available data from the above datasets for a random sample of global cities, stratified by population class, GNI, and ecoregion. Selection was weighted to preferentially select cities with the most data already held within existing global datasets. The database was then supplemented with data from federal and city-level datasets, and data derived from some of the above geospatial and climate datasources. The database has been used in ‘epidemiology of the urban’-type analyses, to investigate inter-city variations in heat exposure due to climate change [82], and environmental risks [83].

However, the above obstacles meant that the process of combining datasets was not straightforward, emphasising the need for consistent, standardized data on city characteristics, including environmental exposures, population behaviours, and health, to provide the underlying empirical data which may be used to evaluate sustainability and health performance of cities and policies, and to track progress towards SDGs. A greater investment in data collection, using an extended set of standardized statistics—including health information—would provide valuable data for such analysis. This is not likely to be possible to do on a global scale, but could be done for a network of sentinel cities selected to be globally representative—using, for example, the approach of the AoE or the UN Sample of Cities. Combinations of standardised statistics, data acquired from satellite imagery and sensor networks, and data aggregated from health information systems may form the basis of future datasets, particularly as access to such technology becomes more widely available.

## 5 Conclusions

We evaluate the feasibility of combining existing datasets to provide a basis for empirical analyses of how city characteristics relate to sustainability and health. An initial scoping review of available datasets and organisations related to health and sustainability in global cities is performed, identifying key global initiatives and summarizing their scope, number of cities, and the availability of their data. Analysis of the cities included in the various initiatives indicates that cities rarely have globally representative distributions with respect to wealth, population size, and ecoregion classifications. We show that smaller cities are underrepresented in most databases, which therefore complicates efforts to monitor environmental, economic and health data in rapidly growing cities, particularly in sub-Saharan Africa where population growth is highest. We also demonstrate the poor overlap between the datasets, which can limit the scope for aggregating data into a single representative sample. There are several obstacles in aggregating such data, including issues with coverage, data quality, reliability, and accessibility; key issues include a lack of standardisation and often limited overlap of cities represented in different databases, which makes it difficult to assemble integrated data for research. Health data are often not reported at city level, which adds to the complexity of quantifying the health impact of changes in environmental exposures or health-related behaviours.

Comparative data are important to help understand opportunities for improving sustainability and public health through city policies, but such comparisons need to take account of factors such population size, climate/ecoregion, and wealth. Quantitative data on cities enables greater understanding of the characteristics of sustainable, healthy cities, and may help inform city-specific policies. Greater efforts should be made to establish city-wide surveillance systems, particularly in low-income settings, to collect essential data relevant to the monitoring of the SDGs and other development priorities.

## Figures and Tables

**Table 1:** Search terms for scoping review. Topic searches within the articles title, abstract, and keywords.

Topic=		Sustain *		Database			City			Global	
						
		Health *		OR	Dataset			cites		OR	International
		Environment			Organi * sation			Urban			
	OR	Transport *	AND		Association	AND					
		Housing			Index		OR		AND		
		Energy			Indices						
		Diet			Indexes						
		Policy									
		Demograph *									

An asterisk (*) is used for an unknown or “wildcard” text.

**Table 2:** International city networks and coordinating organisations. Partnerships and associations are denoted by superscript letters. Weblinks to the organisations may be found in the references.

	Organisation	Number of Cities	Scope	Reference
Networks	100 Resilient Cities ^c^	100	Resilience to physical, social, and economic challenges	[20]
	Age Friendly Cities	499	Knowledge exchange on meeting needs of elderly residents	[21]
	C40 ^a,b,d,f,i^	94	Climate change, GHG emission and climate risk reduction, urban health, wellbeing, and economic opportunity	[18]
	Carbon Neutral Cities Alliance (CNCA) ^d^	20	Achieving carbon reduction goals	[22]
	Carbon Disclosure Project (CDP) ^a,f^	572	Measuring and understanding environmental impact	[17]
	Commonwealth Local Government Forum (CLGF) ^e,i^	200	Support development of democracy and good governance	[23]
	Compact of Mayors ^f^	596	Greenhouse gas and climate risk reduction	[15]
	Covenant of Mayors ^f^	7744		[14]
	Global Cities Indicators Facility (GCIF)	256	Collection of standardized city metrics	[19]
	Global Covenant of Mayors for Climate and Energy (GCMCE) ^f^	7499	Climate leadership	[15]
	International Council for Local Environmental Initiatives (ICLEI) ^b,f,g,h,i^	1227	Sustainable development	[24]
	Organization of Islamic Capitals and Cities ^g^	141	Sustainable development	[25]
	Safer Cities ^l^	77	Improve urban safety and reduce crime	[26]
	Sister City International	>2000	Municipal cooperation, cultural understanding, and economic development	[27]
	The World Association of Major Metropolises (Metropolis) ^f,g,I,j^	137	Collaborative projects and learning	[28]
	Global Compact Cities Programme (GCCP) ^l^	109	Collaboration between government, civil society, and the private sector	[29]
	United Cities and Local Governments (UCLG) ^f,I,k^	<1000	Promote the values, objectives, and interests of local governments through cooperation	[30]
	World Mayors Council on Climate Change (WMCCC) ^h^	80	Advocate for increased role of local governments in climate change mitigation	[31]
Coordinators	Cities Alliance ^c,e,f^		Group of Local government associations	[32]
	Global Taskforce for Local and Regional Governments ^i^		Coordinates major international networks of local governments	[33]
	International City/County Management Association		Developing and foster professional management in cities	[34]
	International Network for Urban Development ^j^		Association of urban policy-makers and practitioners	[35]
	UN Advisory Committee of Local Authorities ^k,l^		Advisory body to strengthen dialogue between UN and local authorities	[36]
	World Urban Campaign ^l^		Advocacy and partnership to create green, productive, safe, healthy, inclusive, and well-planned cities	[37]
	UN Habitat ^l^		Promote socially and environmentally sustainable human settlements	[38]

**Table 3:** Sample of datasets with data on global cities on the themes of health and sustainability.

	Database	Number of Cities	Scope	Data Collection Method	Availability	Reference
**Derived**	Atlas of Urban Expansion (AoE)	200	Urban form, density, and roads	Satellite imagery	Public	[55,56]
	Guo et al.	305	Temperature-related mortality	Health records and ambient temperature data	Academic subscription	[49]
	Bulkeley et al.	94	Database on climate change experiments	Scoping study	Academic subscription	[54]
	Lelieveld et al.	20	Premature mortality due to air pollution	Health modelling study	Academic subscription	[59]
	Schneider et al.	141	Urban land use	Satellite imagery	Academic subscription	[57]
	ShareCity100	98	Food sharing	Landscape analysis	Academic subscription	[50]
	Wang et al.	25	Energy Efficiency	Derived from Global Power City Index (GPCI)	Academic subscription	[60]
	Pareekh et al.	37	Transportation diversity	Exploratory factor analysis	Academic subscription	[61]
	International Physical Activity and Environment Network (IPEN) Adult Study	14	Physical activity and urban attributes	Cross sectional study and GIS analysis	Academic subscription	[51]
	Cheng et al.	71	Ambient PM_2.5_ concentration	Comparison of reported monitored data	Academic subscription	[53]
	Karagulian et al.	320	City source apportionment of PM_10_ and PM_2.5_	Systematic Review	Academic subscription	[52]
**Aggregated/Reported**	Brookings Global Metro Monitor	300	Economic growth	Aggregated from individual city data	Public	[62]
	C40	94	Climate change, GHG emission and climate risk reduction, urban health, wellbeing, and economic opportunity	City government reporting	Public	[18]
	Carbon Disclosure Project (CDP)	500	Impact of cities on the environment	City government reporting	Public	[17]
	Geonames	23,344	Population, location for cities over 15,000 population	Multiple sources, including National Geospatial-Intelligence Agency’s (NGA) and the U.S. Board on Geographic Names	Public	[11]
	NY City Global City Data	90	Geography, topography, demographic, economic, environmental, health, and cultural indicators	Aggregated from individual city data	Public	[43]
	OECD Metropolitan Areas	282	Demographics, economy, land use, quality of life	Government reporting	Public	[42]
	UN Data Explorer	245	Demography, economy, resilience, transport, health, education, crime, and topography	Local government reporting, local urban observatories	Public	[41]
	UN Office for Drugs and Crime (UNOCD)	128	City crime (homicide)	National and international policing agencies	Public	[63]
	UN Sample of Cities	200	Urban characteristics including environment, pollution, transport, and housing	Satellite imagery, government, and local observers	Public	[56]
	UN Urban Data	741	Population, resilience, wealth, transport, health, education, crime, land area	Urban statistics compiled by UN-Habitat’s Global Urban Observatory from household surveys and censuses conducted by national statistics authorities.	Public	[39]
	UN UrbanInfo	604	Housing, demography, communication, energy, economy, education, health, nutrition, disaster, crime, migration, income inequalities, and transport	Urban indicators compiled by UN-Habitat’s Global Urban Observatory from household surveys and censuses conducted by national statistics authorities. Overlaps with UN Urban Data.	Public	[40]
	WHO Ambient Air Pollution	2972	Air Pollution	Local monitoring stations	Public	[44]
	World Bank Urban Transport Data Analysis Tool (UT-DAT)	91	Urban Transport	City government reporting	Public	[45]
	World Council on City Data (WCCD)	30	Comprehensive standardised set of 100 indicators (including 46 core)	City government reporting	Public	[46]
	World Urban Database	99	Urban landcover and local climate zones	Volunteer remote sense data analysis	Public	[64]

**Table 4:** The number of shared cities between the different databases. Refer to Table 3 for acronym definitions and details.

	Atlas of Urban Expansion	Brookings	CDP/C40	NY Cities Database	OECD	UN Data	UN Habitat	UN ODC	UN UrbanInfo	UT-DAT	WCCD	WHO
**Atlas of Urban Expansion**		51	27	38	34	138	93	32	57	35	6	57
**Brookings**	51		67	73	150	211	207	43	62	64	15	175
**CDP/C40**	27	67		44	53	72	68	28	24	35	10	48
**NY Cities Database**	38	73	44		47	78	79	40	37	50	14	52
**OECD**	34	150	53	47		207	125	25	33	40	10	132
**UN Data**	138	211	72	78	207		588	114	348	85	19	754
**UN Habitat**	93	207	68	79	125	588		92	300	74	19	261
**UN ODC**	32	43	28	40	25	114	92		63	30	7	53
**UN UrbanInfo**	57	62	24	37	33	348	300	63		43	9	127
**UT-DAT**	35	64	35	50	40	85	74	30	43		13	67
**WCCD**	6	15	10	14	10	19	19	7	9	13		14
**WHO**	57	175	48	52	132	754	261	53	127	67	14	
